# Natural Frequency Response Evaluation for RC Beams Affected by Steel Corrosion Using Acceleration Sensors

**DOI:** 10.3390/s20185335

**Published:** 2020-09-17

**Authors:** Yuwei Zhang, Yongchun Cheng, Guojin Tan, Xiang Lyu, Xun Sun, Yunshuo Bai, Shuting Yang

**Affiliations:** College of Transportation, Jilin University, Changchun 130025, China; ywzhang@jlu.edu.cn (Y.Z.); chengyc@jlu.edu.cn (Y.C.); lvxiang18@mails.jlu.edu.cn (X.L.); sunxun18@mails.jlu.edu.cn (X.S.); baiys19@mails.jlu.edu.cn (Y.B.); yangst18@mails.jlu.edu.cn (S.Y.)

**Keywords:** steel corrosion, natural frequency, RC beam, piezoelectric acceleration sensors

## Abstract

This paper presented a laboratory investigation for analyzing the natural frequency response of reinforced concrete (RC) beams affected by steel corrosion. The electrochemical acceleration technique induced the corroded RC beams until the predetermined value of the steel corrosion ratio was achieved. Then, the natural frequency responses of the corroded beams were tested utilizing piezoelectric acceleration sensors. The damage states of the corroded beams were assessed through the measurement of crack parameters and the equivalent elastic modulus of the beams, which aims to clarify the fundamental characteristics of the dynamic response for the corroded RC beam with the increased steel corrosion ratio. The results revealed that steel corrosion reduces the bending stiffness of the RC beams and, thus, reduces the modal frequency. The variation of natural frequency can identify the corrosion damage even if no surface cracking of the RC beam, and the second-order frequency should be more indicative of the damage scenario. The degradations of stiffness and the natural frequency were estimated in this study by the free vibration equation of a simply supported beam, and a prediction method for the RC beam’s residual service life was established. This study supports the use of variations in natural frequency as one diagnostic indicator to evaluate the health of RC bridge structures.

## 1. Introduction

The existing reinforced concrete (RC) structures need regular maintenance during the in-service period, even so, most of the RC structures do not reach their design life. The reason is the lack of durability [1,2]. Nowadays, engineers and researchers have recognized that steel corrosion is a major factor that deteriorates the durability of RC structures [3,4,5]. In the past, the method for corrosion assessment of steel bars embedded in the RC structure is destructive (such as the steel mass loss measurement after core sampling [6,7]), which is harmful and uneconomical to the RC structures [8,9]. This problem motivated the interest in utilizing nondestructive techniques for structural health monitoring; and the main purpose of structural health monitoring is to detect structural damage and reduce the risk of structural failure which may lead to casualties and property losses.

Nowadays, nondestructive testing methods (including using strain gauges [10] and optical fiber sensors [11]) are widely used for evaluating the mechanical properties of RC structures. Additionally, using piezoelectric acceleration sensors for structural damage identification has become a popular technology for monitoring the health status and the quality acceptance of RC constructions [12]. Cho et al. [13] used piezoelectric acceleration sensors to monitor the health of cable-stayed bridges. Hasni et al. [14] detected the damage of the steel frame structure using piezoelectric acceleration sensors and found that the sensors could effectively detect and locate the crack and bolt loosening. Sung et al. [15] used a variety of methods to monitor the bridge structure and verified the accuracy of the acceleration sensor in structural health monitoring. The natural frequency, modal shape, and modal damping are the inherent properties in an RC structure, which are the functions of mass, damping, stiffness, and boundary conditions [16,17,18,19]; and the natural frequency is also an integral characteristic of one RC structure and any changes in the natural frequency indicate a variation in the structural characteristic, such as damage or strengthen. Mu et al. [20] optimized the uncertainty of modal frequency under the influence of environmental factors. Park et al. [21] found that the fractional-order decreasing rate of modal frequency decreases with the increase of modal number, but the absolute decreasing rate increases. Therefore, monitoring the natural frequency indicators is a simple and convenient method that could provide a good reference for describing the overall structural status [22].

At present, the health monitoring of existing RC structures with steel corrosion has gradually attracted people’s attention [23,24]. However, most researches focus on the identification of structural steel-corrosion-induced cracking damage. There is little literature that illustrates the effect of steel corrosion on the frequency response of RC structures. Lin et al. [25] studied the frequency variation of RC under accelerated corrosion conditions. They found that the natural frequency of the RC beam decreases with the increase of steel corrosion rate or the decrease of concrete resistivity. Heitner et al. [26] studied the relationship between bridge health monitoring and steel corrosion in bridges. Razak et al. [27] studied the modal parameters of RC beams under the influence of steel corrosion and concluded that damping is an unreliable corrosion damage parameter of RC beams, while the natural frequency can effectively reflect the structural capacity changes caused by the steel corrosion in the test beams. Koh et al. [28] and Capozucca et al. [29] illustrated that the damage of the RC structure caused by steel corrosion will inevitably lead to the change of performance such as modal frequency, stiffness, damping ratio, and so on. However, these researches did not analyze the sensitivity of natural frequency response to the steel corrosion, and it is unknown whether the occurrence of steel corrosion can be identified even if no cracks can be observed on the surface of the structure. Therefore, although the use of natural frequency as a health assessment parameter is simple and popular, it still needs to further study the natural frequency response in the corrosion detection area.

Thus, utilizing the piezoelectric acceleration sensors, this paper is concerned with the natural frequency response of the RC beam under the effect of steel corrosion. The effectiveness of the identification of steel corrosion was analyzed based on the natural frequency response. Besides, the mechanism of the reduction of the natural frequency response of corroded RC beams is clarified by analyzing the equivalent elastic modulus. This study also established a prediction method for structures’ residual service life, which may provide the engineers with better insight into the health assessment of RC structures.

## 2. Materials and Experiments

### 2.1. Materials and Mixture

HRB 335 (tensile strength is 300 MPa) steel bars with a diameter of 8 mm and the length of the 1950 mm were utilized in this study. 

Besides, the average line density (mass per meter) of all the steel bars should be tested, and the test method will be illustrated below. PO 42.5 type Portland cement was used in this study. Crushed stones were used as coarse aggregates, and the diameters ranged from 2.36 to 20.0 mm. Besides, the natural sands were used as fine aggregates, and the fineness modulus was tested to be 2.9. The mixture proportions of concrete are listed in Table 1.

### 2.2. Specimen Preparation

**Figure 1:**
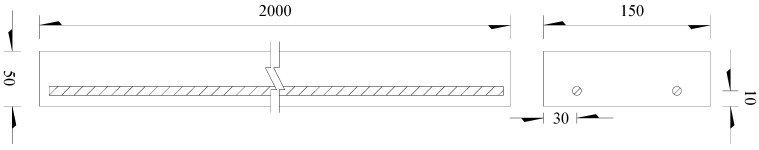
Front view and lateral view of reinforced concrete (RC) beam specimen (unit: mm).

The RC beam specimens prepared for this study composed of concrete and two steel bars. Dimensions of the specimens and relative positions of the two steel bars are shown in Figure 1.

The concrete mixtures were prepared in the laboratory by a pan mixer. Beams with a size of 2000 × 150 × 50 mm^3^ were cast in wooden molds. Then, they were allowed to cure at the conditions of 20 °C and 95% relative humidity and removed from the molds after 24 h curing. After that, all the RC beams were cured at the same conditions for another 28 d.

### 2.3. Experimental Methods

#### 2.3.1. Scheme Design

**Figure 2:**
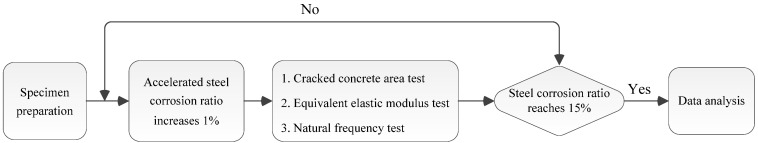
Experimental procedure.

Two RC beam specimens were used in this study to reduce the experimental error. After curing, an electrochemical accelerated steel corrosion experiment was performed to get the corroded beams for investigating the influence of steel corrosion on the natural frequency response. Besides, during the steel corrosion process, the concrete cover must be cracked due to the expansion of stress. Thus, the damage states of the corroded beams should be assessed through the measurement of crack parameters (width and length) and the equivalent elastic modulus of the beams. Besides, the natural frequencies of RC beams were also needed to test, which aims to clarify the fundamental characteristics of the dynamic response for the corroded RC beam with the increased steel corrosion ratio. The experimental procedure was determined and shown in Figure 2.

Firstly, two RC beams were used in an electrochemical accelerated steel corrosion experiment for getting the corroded steel bars. The theoretical maximum steel corrosion ratio was controlled to be 15%. Subsequently, for the steel where the corrosion ratio increases every 1%, the widths and lengths of the steel-corrosion-induced cracks, natural frequencies, as well as equivalent elastic modulus of the two RC beams, should be tested until the steel corrosion ratio reaches 15%. Finally, the tested data will be used for further analyzing and achieving the research targets of this paper.

#### 2.3.2. Electrochemical Accelerated Steel Corrosion 

**Figure 3:**
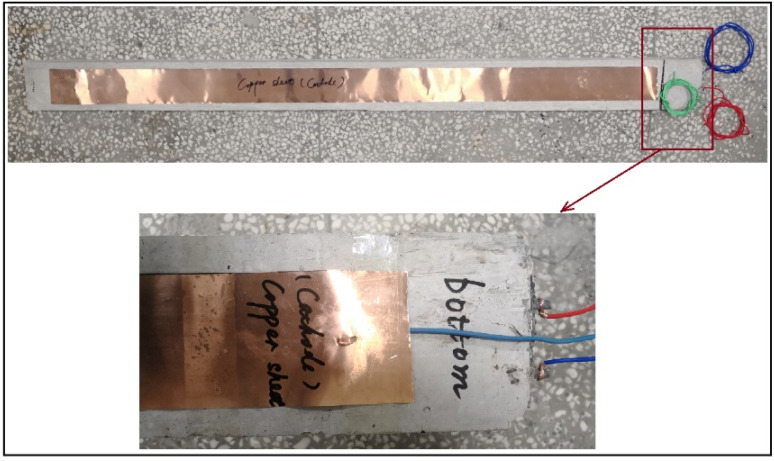
Connection schematic diagram of the copper sheet and the copper wires on the RC beams: copper sheets welded with the baby blue and green skin copper wires; and steel bars welded with dark blue and red skin copper wires.

After cured, concrete at the end of the RC specimens within a range of 10 cm was cut by an angle grinder for exposing part of the steel bar; and a copper sheet was tied to the bottom of each specimen. As shown in Figure 3, exposed parts of the four steel bars in two RC beams were welded to the copper wires with dark blue and red skin. Besides, the two copper sheets on the bottom of the two RC beams were welded to the copper wires with baby blue and green skin, respectively.

In general, it will take a long time to observe the corrosion of steel bars by immersing RC specimens into chloride solution due to the slow penetration process of chloride ions. The electrochemical technique shows an effective way to accelerate the steel corrosion process, which is popular for scholars in recent researches [30].

According to our previous study [9], before specimen preparation, all the steel bars were polished with emery paper to dissolve the rust, then ethanol and acetone treatments were used to degrease their surfaces. Subsequently, steel bars should be cleaned with clear water and then dried at 20 °C for 4 h. After that, the mass and length of each steel bar should be tested for obtaining the average line density of all the steel bars. The value of the average line density of steel bars was tested to be 0.366 kg/m.

Furthermore, to determine the theoretical current value and electrified duration, Faraday’s law was used to calculate the theoretical steel mass loss of steel bars embedded in each specimen when subjected to accelerated corrosion using impressed current. The relation between the steel mass loss and current intensity is as follow:

(1) $$\Delta m=\frac{Mit}{zF},$$

where Δm is theoretical steel mass loss, in g; *M* is the atomic weight of the steel bar (56 g); i is impressed current intensity, in A; t is corrosion duration, in s; z is the ionic charge, which is equal to 2; F is Faraday’s constant (96500 A/s).

In this study, the maximum corrosion ratio was 15%. Comprehensive considering the test process and the maximum current value, a 30 V direct current (DC) regulated power supply was used to provide impressed current. The time for the accelerated steel corrosion ratio to increase by 1% was controlled to be 24 h. So, the applied current value determined from Faraday’s law to reach the theoretical corrosion ratio is shown in Table 2.

It was ensured that the corrosion ratio of the four steel bars embedded in two RC test beams was the same. Thus, to shorten the experiment duration, the four steel bars should be connected in parallel with the DC regulated power supply, and the impressed current value provided by the power supply is 1.12 A; the electrical accelerated steel corrosion was carried out for 15 d.

**Figure 4:**
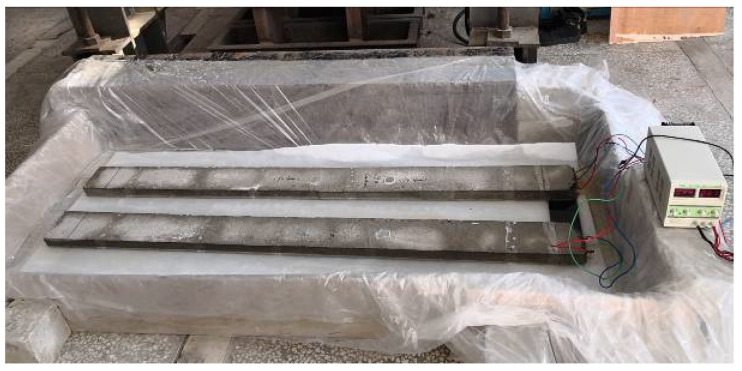
Experimental setup for accelerated steel corrosion.

Besides, as shown in Figure 4, the DC regulated power supply was used to provide impressed current, and two RC beams were placed in an electrolytic tank filled with 6 wt% NaCl solution. Four steel bars which in two RC beams were connected to the anode of DC regulated power supply in parallel mode, while the copper sheet which acted as the cathode was connected to the cathode of DC regulated power.

Besides, before the accelerated steel corrosion test, specimens should be immersed into NaCl solution for 2 d to ensure the chloride ions can penetrate into concrete under capillary action. Meanwhile, during the accelerated steel corrosion test, NaCl solution should not touch the exposed part of steel bars.

#### 2.3.3. Test Method for the Natural Frequency Response 

**Figure 5:**
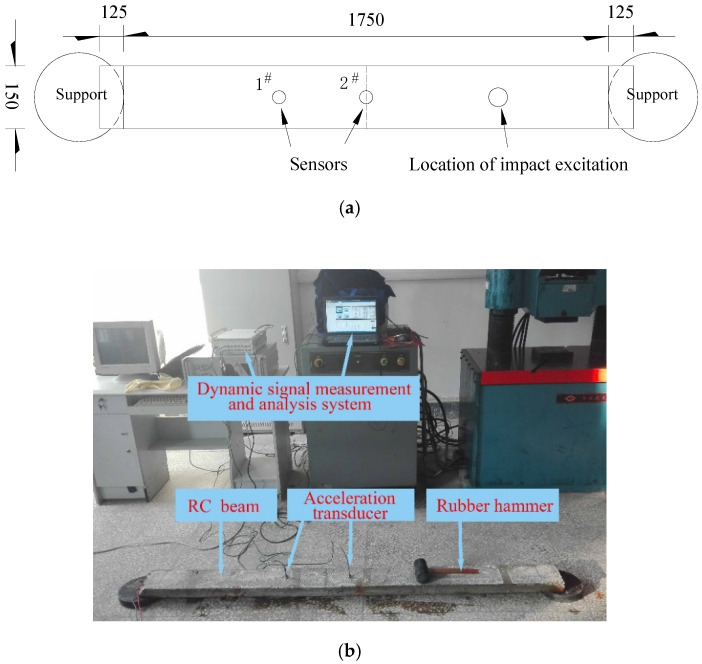
Natural frequency response test: (**a**) sketch for supports and sensors locations (unit: mm); (**b**) experimental setup for the natural frequency response test.

To achieve a more accurate frequency result, two disk shape rubbers were used as supports, and the edge of the disk shape rubber was tangent to the support line of the RC test beam. Two DH131E piezoelectric accelerometers (produced by Jiangsu DongHua Testing Technology Co., Ltd., TAIZHOU, JNG, China) were used to obtain the acceleration response under impact excitation from a rubber hammer at a 1/4 span length from the right end. The 1^#^ accelerometer was placed at a 1/3 span length from the left end, and the 2^#^ accelerometer was placed at midspan. A DH5922 type dynamic signal measurement and analysis system (produced by Jiangsu DongHua Testing Technology Co., Ltd., TAIZHOU, JNG, China) were used to measure and analyze acceleration response. The setup for the natural frequency response test is shown in Figure 5.

**Figure 6:**
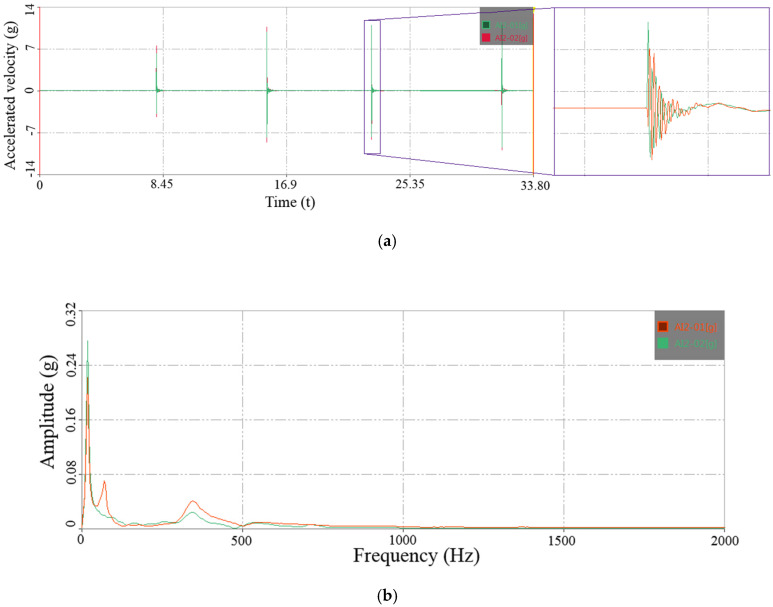
Acceleration time history and corresponding amplitude spectrum: (**a**) acceleration signal history; (**b**) amplitude spectrum.

DHDAS-2013 software platform performed data processing. The Hamming window was used to intercept the acceleration signal. The number of spectrum lines was set to 6400, and the obtained frequency resolution was 0.313 Hz. Natural frequencies were identified and extracted by frequency spectrum analysis using the fast Fourier transform (FFT). During one frequency response test, at most four-time impacts were applied, and the rest time between two impacts was controlled to 8 s. Besides, during the frequency response test, the impact force must be applied, which must lead to some damage to the RC beam. Therefore, in order to reduce the destructive effect of impact force on the RC beam, the height of the rubber hammer and beam was controlled at 30 cm. Under the premise of making the bridge vibrate, the impact force should be kept as small as possible. Thus, it can be assumed that the impact force will not elongate the cracks and aggravate the damage of the RC beam. Unfortunately, due to the limitations of the experimental equipment, each applied impact force was not able to be measured during the natural frequency response test process. As an example, representation data from one RC specimen collected from the piezoelectric acceleration sensors are shown in Figure 6; both the time history of the acceleration signal and the corresponding amplitude spectrum are illustrated.

#### 2.3.4. Test Method for Cracking Area

Steel corrosion causes the expansion of the steel bar, which exerts pressure and cracks the surrounding concrete. A large number of studies have shown that cracks degrade the mechanical properties of concrete materials. Therefore, it is important to clarify the relationship between the crack and the dynamic properties of RC beams for further analyzing the RC beam’s natural frequency response affected by steel corrosion.

Each steel-corrosion-induced crack has three parameters: length, width, and depth. For these three parameters, only the length and width of the crack need to be measured due to the depth of the steel-corrosion-induced crack almost equaling the thickness of the concrete cover. The experimental phenomenon showed that there formed multiple steel-corrosion-induced cracks along the steel bars at the bottom of each RC beam, and most of the cracks eventually connected with each other. Therefore, the cracks formed by one corroded steel bar can be set into one group, a total of 4 groups in the two RC beams; thus, each group contains multiple cracks. Crack width was detected using a crack width recorder, and the equivalent crack width was decided as the average of measurement values from 4 measured points on each crack. Besides, it is hard to measure the crack length accurately for the irregular direction of crack extension, so the projected length of the crack along the length of the test beam should be measured and decided as the equivalent length of the crack. Therefore, the area of each crack can be calculated by multiplying the equivalent length by the equivalent width, and the cracking area of each RC beam is the sum of all cracking areas in the two groups of cracks on the beam.

#### 2.3.5. Test Method for Equivalent Elastic Modulus

Elastic modulus is an inherent property of one material, and it is one of the important indexes to measure the degree of elastic deformation. Besides, the elastic modulus is one of the parameters that determine the vibration behavior of the RC beam. Therefore, it is necessary to clarify the relationship between the steel corrosion and the equivalent modulus of elasticity of RC beams for further analyzing the RC beam’s natural frequency response affected by steel corrosion.

Concrete is an anisotropic material composed of mortar and aggregates. However, the concrete material is usually assumed to be an isotropic material with a single modulus of elasticity during the bridge design process, and also considers the contribution of the steel bar to the flexural stiffness of the bridge section; this simplification is effective. Therefore, this paper assumes that the reinforced concrete material is isotropic. Besides, flexural stiffness is an index that determines the maximum bearing capacity of an RC beam, and thus this paper aims to study the effect of steel corrosion on the equivalent flexural elastic modulus (equivalent flexural stiffness) of the beam.

In general, it is difficult to directly measure the flexural elastic modulus of composite materials such as RC materials or bridge superstructure, in this study, the equivalent elastic modulus of RC beam was obtained by applying static load and measuring the midspan deflection of the RC beam.

**Figure 7:**
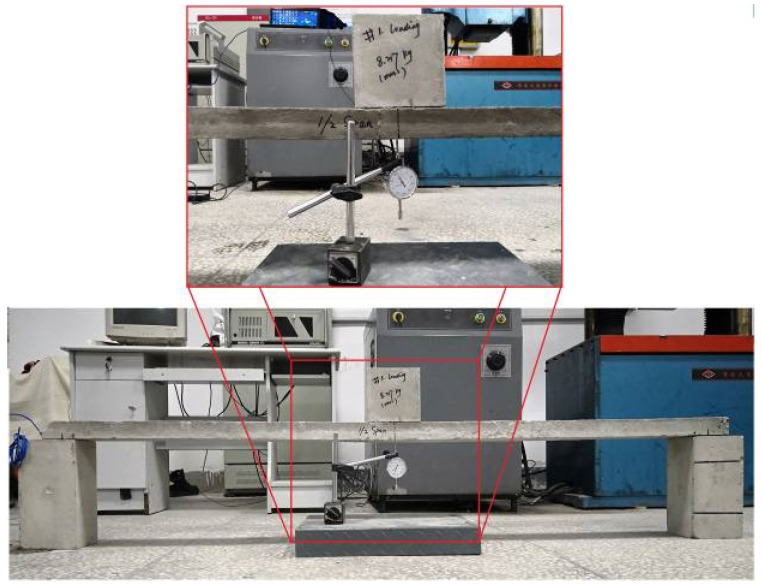
Setup for equivalent elastic modulus test.

Although the static load is harmful to the damaged RC beam and it may elongate the crack, it is an effective method that could reflect the flexural stiffness of the RC beam through the measurement of the deflection. Therefore, on the premise of ensuring the observable deformation of the bridge, the added mass (treated as static load) should be as small as possible. In order to determine the added mass, we had performed a preliminary experiment. We found that when the added mass is less than 10 kg, even if the steel corrosion ratio reaches 15%, the crack width can remain constant before and after the static load test. Therefore, a cubic concrete with a weight of 8.217 kg was chosen and placed on the midspan of the RC beam for exerting constant load. The setup for measuring the equivalent elastic modulus of the RC test beams is shown in Figure 7.

The distance between two supporting points at the bottom of the RC beam was controlled to be 175 cm. A dial gauge was arranged at the bottom of the midspan of the RC beam. Thus, the deflection can be obtained by reading the value of the dial gauge before and after exerting the constant load. Besides, since the side length of the cubic concrete is relatively small compared with the length of the RC beam, the constant load can be treated as a concentrated load. Thus, the relationship between the deflection (*Y*) of the simply supported beam and the equivalent elastic modulus is as follows:

(2) $$Y=-\frac{FL^{3}}{48EI},$$

where *F* is the constant load, 80.53 N; *L* is the span length of RC beam, 1.75 m; *E* is the equivalent modulus of elasticity, in Pa; *I* is the moment of inertia of the rectangular section, which can be calculated according to the dimension of RC beam described in Figure 1, in m^4^.

## 3. Results and Discussions

### 3.1. Effect of Steel Corrosion on the Natural Frequency Response of RC Beams

**Figure 8:**
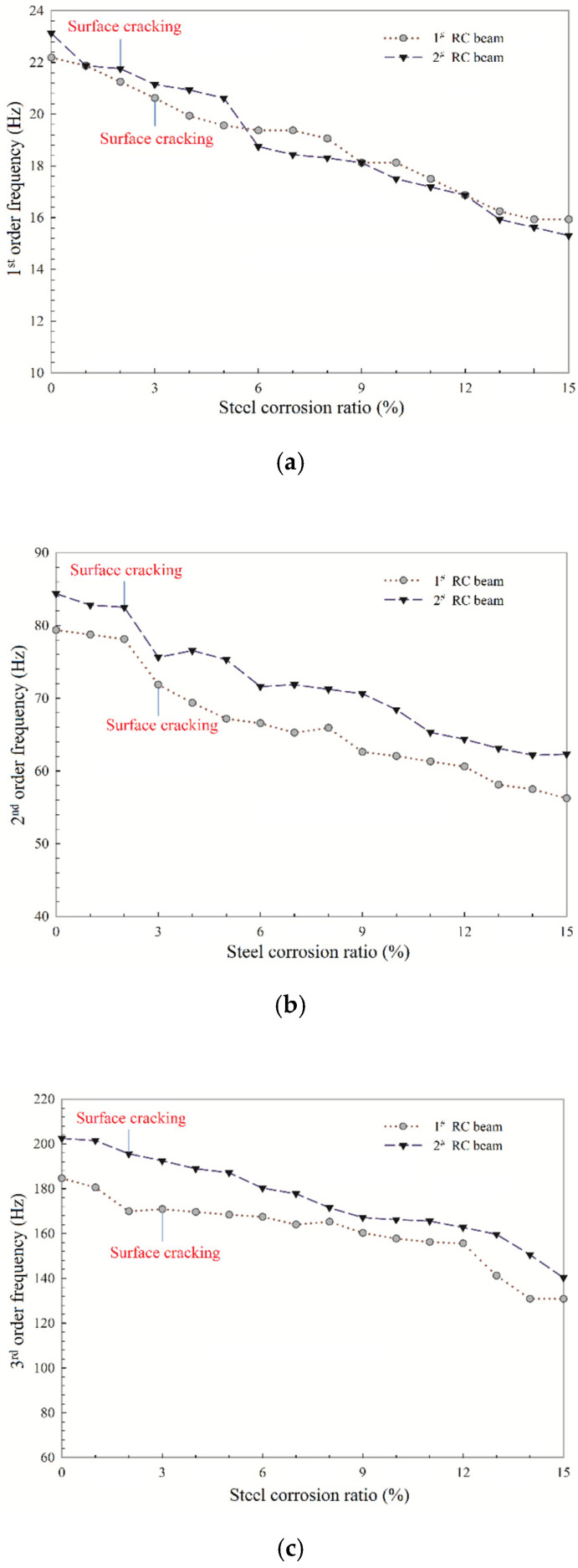
Effect of steel corrosion on natural frequencies of the two RC beams: (**a**) first-order frequency; (**b**) second-order frequency; (**c**) third-order frequency.

The test results of the first three order natural frequency for the two RC beams are shown in Figure 8.

From Figure 8, it can be seen that once the steel corrosion process advances, the first three order frequencies decrease in a similar way in all the RC beams. Besides, along the process of the experiment, cracks can be observed on the surface of concrete when the steel corrosion ratio reaches 4% for the 1^#^ RC beam, and 3% for the 2^#^ RC beam, respectively. Before concrete surface cracks occurred, the natural frequencies of the two RC beams are declining, indicating that steel corrosion has caused some damage inside the concrete before the cracks appeared on the concrete surface. Besides, it can also be seen that all the three order natural frequencies keep decreasing with the increase of steel corrosion after concrete surface cracking. Furthermore, the occurrence of concrete surface cracks significantly decreases the second-order frequencies. In general, higher modal frequencies are sensitive to an incipient crack formed in RC beams than lower ones [31]. The experimental results in this section reveal that second-order frequency is sensitive to the formation of steel-corrosion-induced cracks on the concrete surface, which may be due to the low spectral power of the acceleration signal at the third-natural frequency, thus, less accurate measurement. Thus, considering the high order frequency values are difficult to obtain accurately, this paper supports the use of second-order frequency as an index for evaluating the corrosion damage of the bridge and monitoring the bridge structures’ surface cracking states more conveniently. Moreover, it is obvious that as the corrosion ratio of the steel bar in the 2^#^ RC beam reaches 5–6%, the first and second order frequencies decrease sharply. It can be explained from the experimental phenomenon that during the process of the steel corrosion ratio changes from 5% to 6%, the width of cracks along the length direction of the bridge barely changed, while the length of the cracks expanded significantly. This indicates that the expansion stress caused by the corrosion of steel bars is fully released during this process and deteriorates the concrete properties of the RC beam. The same situation also occurred at the corrosion ratio changed from 8% to 9% in the 1^#^ RC beam, which means that the decrease of the natural frequency response is a sign of internal damage to the RC structures.

### 3.2. Effect of Steel-Corrosion-Induced Crack on the Natural Frequency Response of the RC Beam

**Figure 9:**
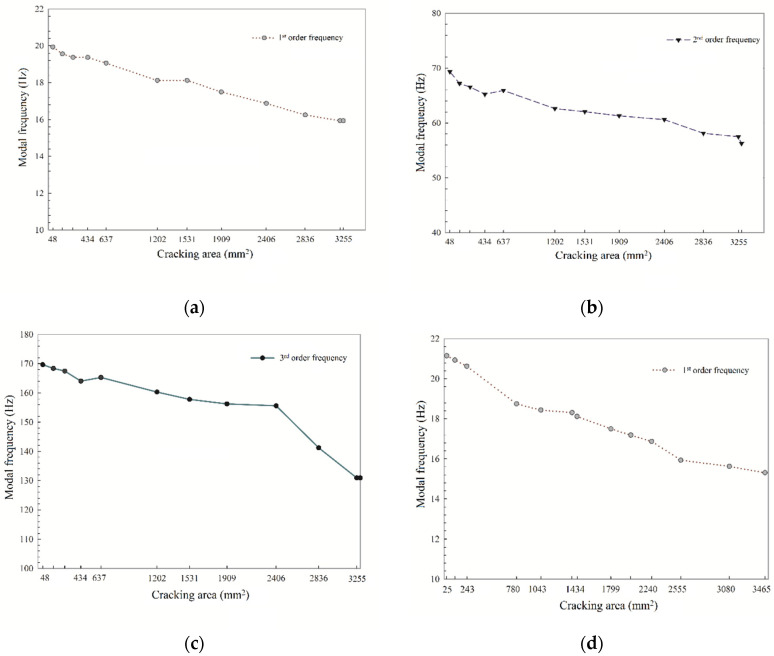

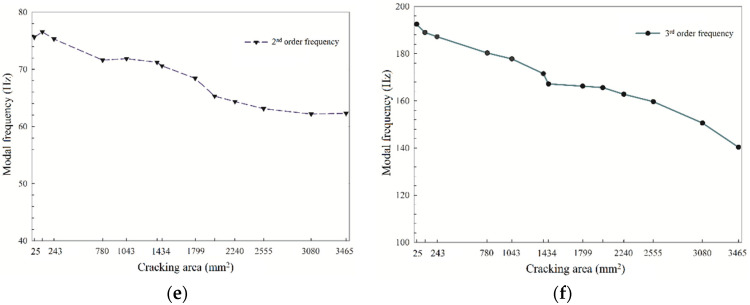
First three order natural frequencies of the two RC beams affected by cracking area: (**a**) first-order frequency of the 1^#^ beam; (**b**) second-order frequency of the 1^#^ beam; (**c**) third-order frequency of the 1^#^ beam; (**d**) first-order frequency of the 2^#^ beam; (**e**) second-order frequency of the 2^#^ beam; (**f**) third-order frequency of the 2^#^ beam.

In general, steel-corrosion-induced crack is unavoidable during the process of steel bar corrosion in the RC structure. However, from Section 3.1, the natural frequencies of RC beams decrease not only before but also after the concrete surface cracking of the RC beams, suggesting that the action of the crack is also a key factor that influences the natural frequency response. Hence, it is necessary to quantitatively evaluate the influence of the crack on the natural frequency response. The cracking areas were determined by the width of crack times the length of each crack, measured on the surface of the concrete. The test results for the cracking area are listed in Table 3 and the relationship between the cracking area and natural frequency response is shown in Figure 9.

The cracking area factor presents the similar variation laws as the steel corrosion process that the first three order natural frequencies of the two RC beams all decrease with the increased cracking areas. Overall, the reasons can be explained from the phenomenon of the experimental process as follows. At the early edge of the corrosion process rust increases the volume of the steel bar and causes expansion stress on surrounding concrete; then, expansion stress exceeds the maximum tensile stress that concrete could resist, and a corrosion-induced crack occurs in several locations. As the corrosion process goes on, cracks are connected and run through along the length direction of the RC beam due to the generally increased expansion stress and strain energy accumulated into the concrete. Cracking action results in considerable damage to concrete, which reduces the bond stress between the steel bar and concrete as well as the properties of RC materials [32].

Interestingly, the natural frequency value rises abnormally at some phases for example the cracking areas change from 270.7 to 433.8 mm^2^ in the 1^#^ RC beam. The reason is that at the edge of 270.7 mm^2^ cracking areas, the rust which cannot be expelled from cracks due to the tiny crack width provides extra pressure, leading to the higher local stiffness and strength. While the cracking areas reach 433.8 mm^2^, the experimental phenomenon that observed from the bottom of the RC beam showed that along with the width and length of the cracks continuing to rise, the rust is insufficient to fill the gaps of the cracks and to expel smoothly, reducing the pressure and local strength. Therefore, it is reasonable to believe that the greater the concrete cracking degree is, the lower the bond force between is, the worse the mechanical properties of the concrete are, and the lower the natural frequency value of the RC beam is. Therefore, it is necessary to further analyze the relationship between the steel corrosion ratio, stiffness of RC materials, and natural frequency response.

### 3.3. Effect of Equivalent Elastic Modulus on Natural Frequency Response and Prediction Model for RC Structures’ Residual Service Life

#### 3.3.1. Effect of Equivalent Elastic Modulus on the Natural Frequency

**Figure 10:**
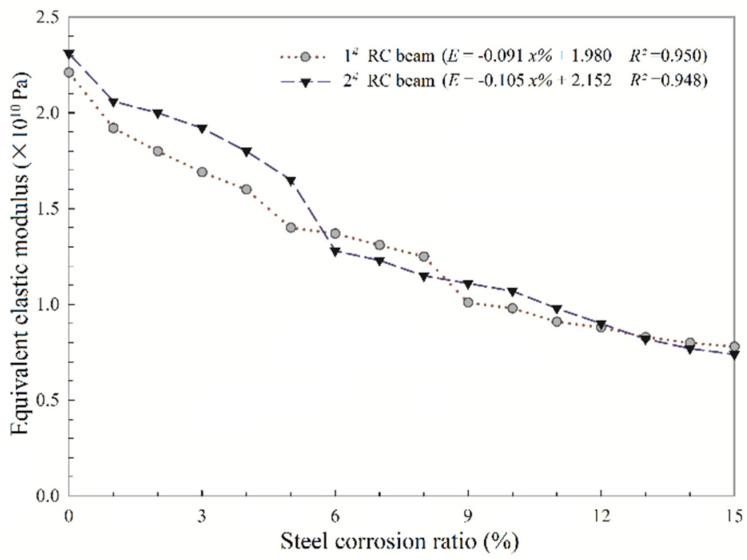
Equivalent elastic modulus data and the regression line models (described in the legends) of the two RC beams affected by the steel corrosion ratio.

The deflection test results are listed in Table 4. The equivalent elastic modulus affected by the steel corrosion of the two RC beams can be calculated by Equation (2), the results are shown in Figure 10.

From Figure 10, it can be observed the equivalent elastic modulus of the 2^#^ RC beam decreases greatly when the steel corrosion ratio reaches 5–6%, while the same phenomenon appears at the corrosion ratio reaches 8–9% of the 1^#^ RC beam, which is the same as the analysis results of natural frequency responses affected by cracking area and steel corrosion ratio. Besides, the equivalent elastic modulus of both the 1^#^ and 2^#^ RC beams tend to decrease with the increase of steel corrosion ratio. Considering that the cross-sectional area of the RC beam has not changed, which indicates that steel corrosion action changes the bond force between steel bar and concrete as well as concrete materials properties, and thus affects the stiffness of the structure. Take the simply supported beam used in this study as the example, the undamped free vibration of the beam at the *n*th order frequency is given by

(3) $$f_{n}=\frac{n^{2}\pi }{2}\sqrt{\frac{EI}{mL^{4}}},$$

where $f_{n}$ is the n th order frequency; *n* is order number; *E* is elastic modulus; *I* is the moment of inertia of the cross-section; *m* is mass per unit length; *L* is the length of span. Therefore, the following equations can be deduced:

(4) $$C=\frac{n^{4}\pi^{2}}{4mL^{4}},$$

(5) $$f_{n}^{2}=C\left(EI\right),$$

Differentiating:

(6) $$2f_{n}\delta \left(f_{n}\right)=C\delta \left(EI\right),$$

(7) $$2f_{n}\delta \left(f_{n}\right)=\left(\frac{f_{n}^{2}}{EI}\right)\delta \left(EI\right),$$

where δ represent the increment of the parameters. Then, the relationship between the increment of stiffness and the *n*th order frequency can be expressed as follows:

(8) $$2\frac{1}{f_{n}}\delta \left(f_{n}\right)=\left(\frac{1}{EI}\right)\delta \left(EI\right),$$

Therefore, the loss in stiffness with changes in natural frequencies can be proposed. As an example, a 10% reduction of the natural frequency would indicate a 20% reduction in the stiffness. Besides, from Figure 10, there is a good linear relationship between the equivalent elastic modulus and the corrosion ratio of steel bars; and the linear equations fitted by the least square method are shown as follows:

(9) $$E_{1}=-0.091x\%+1.980,\;(R^{2}=0.950)$$

(10) $$E_{2}=-0.105x\%+2.152,\;\left(R^{2}=0.948\right)$$

where *E*_1_ and *E*_2_ are elastic modulus of the 1^#^ and 2^#^ RC beam, respectively; *x*% is steel corrosion ratio; *R*^2^ is the correlation coefficient. The high *R*^2^ values indicate this linear model is credible for the experimental results. Then, substituting Equations (9) and (10) into Equation (3), the theoretical *n*th order frequency of the 1^#^ RC beam ($f_{n}^{1}$) and 2^#^ RC beam ($f_{n}^{2}$) can be obtained as follows:

(11) $$f_{n}^{1}=\frac{n^{2}\pi }{2}\sqrt{\frac{I\times \left(-0.091x\%+1.980\right)}{mL^{4}}},$$

(12) $$f_{n}^{2}=\frac{n^{2}\pi }{2}\sqrt{\frac{I\times \left(-0.105x\%+2.152\right)}{mL^{4}}},$$

**Figure 11:**
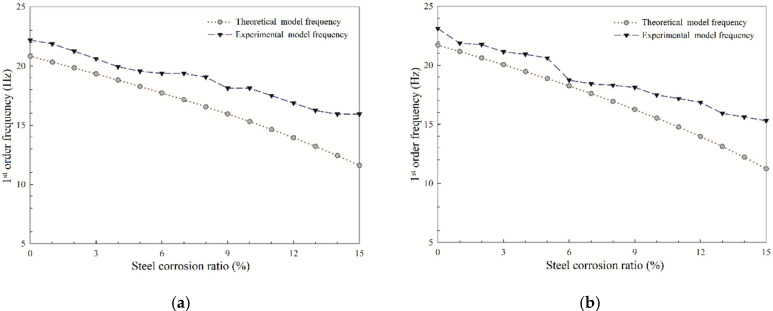
Comparison of theoretical and experimental first-order frequency: (**a**) 1^#^ RC beam; (**b**) 2^#^ RC beam.

Figure 11, as an example, illustrates the comparison between theoretical first-order frequency calculated from Equations (11) and (12) and the experimental results of the two RC beams.

It can be obvious from the figure that both the theoretical and experimental frequency values of the two RC beams are not exactly the same. The reason is the fact that the concrete material is not isotropic while the derived equations in this paper are for the homogeneous beam with isotropic material, which causes the error between the theoretical and experimental natural frequency curves. However, the correlation coefficient values of theoretical and experimental frequency value for 1^#^ and 2^#^ RC beams are calculated to be 0.988 and 0.979, which indicates the natural frequency test results are objective and the model of equivalent elastic modulus change with steel corrosion ratio is effective. This could bring an inspiration that the model of equivalent elastic modulus can be utilized to predict the RC structures’ residual service life more simply and conveniently. Additionally, it can be achieved, as discussed above, that steel corrosion causes the loss to the elastic modulus and the stiffness of the structure before and after the concrete cracking in RC structures, resulting in the reduction of natural frequency response.

#### 3.3.2. Prediction for RC Bridge’s Residual Service Life Based on the Equivalent Elastic Modulus Model

Steel corrosion deteriorates the elastic modulus of the RC bridge and further reduces its natural frequencies. Thus, establishing the model of steel corrosion ratio and RC bridges’ elastic modulus could predict the deterioration of elastic modulus value after measuring the corrosion behavior of steel bars, which could further evaluate and predict the in-service life of RC bridges.

According to our previous studies [8,9], using the electrochemical test methods could measure the corrosion current densities of steel bar which embedded in concrete more exactly; by this, theoretical corrosion volume of steel bar could be obtained after measuring the corrosion current densities of steel bar by

(13) $$\delta_{t}=\frac{Mi_{corr}t}{\rho_{s}nF},$$

where *δ_t_* is theoretical corrosion volume of steel bar, in cm; *M* is the atomic weight of the steel bar (56 g/mol); *i_corr_* is the corrosion current density of steel bar, in *μA/cm*^2^; *t* is corrosion time, in a; *n* is the ionic charge number of steel (2); *ρ_s_* is the density of steel bar (7.86 *g/cm*^2^).

Besides, the corrosion ratio of steel bar can be calculated by

(14) $$x\%=\frac{\delta_{t}}{\Phi_{s}}\times 100\%,$$

where *x*% is steel corrosion ratio; $\Phi_{s}$ is the volume of the steel bar. Therefore, the relationship between steel corrosion ratio and the service time (SCR-T model) of bridge structures can be obtained by Equations (13) and (14).

Therefore, the SCR-T model could provide guidance to predict RC structures’ residual service life. The steps can be described as follows:

Step 1. Obtain the credible equivalent elastic modulus of the undamaged bridge structure (marked as *E*_0_). This parameter could be obtained by numerical simulation based on the bridge’s design information manual; or it could be obtained from static and dynamic load test in-situ, measuring the beam vertical displacement and natural frequencies as illustrated in this paper.

Step 2. Use the electrochemical test method to obtain the corrosion current density (*i_corr_*) of the longitudinal carrying steel bar. Simultaneously, obtain the elastic modulus (marked as *E*) of the bridge structure at the current damage state by static and dynamic load test in-situ. Note that this step should be executed several times within a period (at least once a year) to increase the quality for further prediction. Besides, according to the results in Section 3.3, there may exist a good linear relationship between the equivalent elastic modulus and the steel corrosion ratio; as can be seen from the SCR-T model, the relationship between *i_corr_* and *E* should also be linear. Hence, after obtaining a great number of measurements of *i_corr_* and *E*, the linear model between the *i_corr_* and *E* can be established.

Step 3. To modify the established linear model in Step 2 to be more accurate and reasonable, the value of *i_corr_* and *E* should be continuously measured. However, utilizing the established linear model in Step 2, only measure the *i_corr_* could infer the value of *E*. Therefore, only perform the electrochemical measurements to measure the *i_corr_* value could continue obtaining a great number of measurements of *i_corr_* and *E*, which can greatly reduce the workload of measurement to modify the established model. 

Step 4. Define a% as the elastic modulus loss ratio of RC structures under the ultimate durability state, i.e., it is considered that the bridge failure occurs when the *E* of the bridge is less than *E*_0_ × a%. 

**Figure 12:**
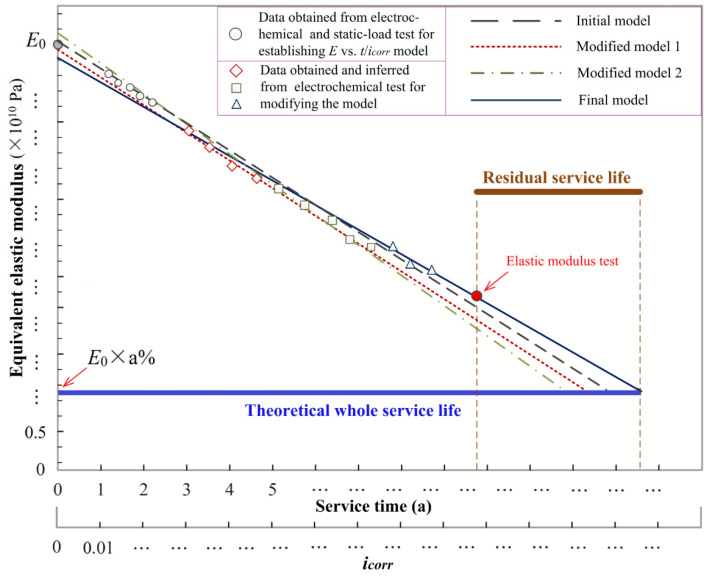
Prediction for RC bridge’s residual service life.

Step 5. The variation of *E* is related to the steel *i_corr_* and the service time (*t*). Hence, taking *E* as the index to predict the bridge’s residual service life can be achieved by establishing the *E* vs. *i_corr_*/*t* diagram. As shown in Figure 12, when the engineers want to predict the bridge’s residual service life, they should measure the equivalent elastic modulus of the bridge in-situ. The corresponding service time can be obtained from the linear model; then, the residual service life can be calculated by the theoretical whole service life (corresponded to *E*_0_ × a%) minus the service time (corresponded to the measurement of *E*).

This prediction method could provide engineers and technicians with a more simple and convenient way to evaluate the RC bridge’s residual service life effectively.

## 4. Conclusions

This study aims to utilize the piezoelectric acceleration sensors for analyzing the natural frequency response of the RC beam under the effect of steel corrosion. After the accelerated steel corrosion test on two RC beams, the natural frequency, cracking area, and equivalent elastic modulus of the test beams were measured, which were used to clarify the mechanism of the reduction of the natural frequency response of corroded RC beams. The following conclusions can be achieved:

(1) Even if no surface cracking of the RC beam, the variation of natural frequency can identify the corrosion damage of the steel bar; and the second-order frequency should be more indicative of the damage scenario especially, which can be used to monitor the bridge structures’ surface crack states more conveniently and accurately.
(2) Steel corrosion reduces the bending stiffness of the RC beams. The trend of the natural frequency values was consistent with the trend observed in the calculated equivalent elastic modulus values from static load tests.
(3) The degradation of stiffness and the natural frequency can be estimated by the free vibration equation of a simply supported beam. Based on this, a prediction method for the RC bridge’s residual service life was established.
(4) The modal frequency obtained by piezoelectric acceleration sensors of the RC structure can be used as a damage indicator for structural properties affected by steel corrosion. The revealed results in this paper support the use of variations in natural frequency as one diagnostic index to evaluate the health of RC bridge structures.

## Figures and Tables

**Table 1:** Mixture proportions of concrete.

Materials	Nominal Proportions (kg/m^3^)
Cement	355
Water	195
Fine aggregate	608
Coarse aggregate	1242

**Table 2:** Accelerated current value for theoretical steel mass loss.

Theoretical Mass Loss	Average Line Density (kg/m)	Mass Loss (g)	Corrosion Duration (s)	Current Intensity (A)
1%	0.366	7.13	86,400	0.28

**Table 3:** Results for cracking area in RC beams.

Steel Corrosion Ratio	Cracking Area in 1^#^ RC Beam (mm^2^)	Cracking Area in 2^#^ RC Beam (mm^2^)
0%	-	-
1%	-	-
2%	-	-
3%	-	25.8
4%	48.0	114.8
5%	155.0	243.6
6%	270.7	780.1
7%	433.8	1043.1
8%	636.6	1379.1
9%	1202.3	1434.5
10%	1530.8	1799.8
11%	1908.7	2014.3
12%	2405.9	2240.0
13%	2835.7	2555.0
14%	3220.0	3080.0
15%	3255.0	3465.0

**Table 4:** Results of deflection and equivalent elastic modulus.

Steel Corrosion Ratio	1^#^ RC Beam	2^#^ RC Beam
	Deflection (mm)	Deflection (mm)
0%	0.26	0.25
1%	0.30	0.28
2%	0.32	0.28
3%	0.34	0.30
4%	0.36	0.32
5%	0.41	0.35
6%	0.42	0.45
7%	0.44	0.47
8%	0.46	0.50
9%	0.57	0.52
10%	0.59	0.54
11%	0.63	0.59
12%	0.65	0.64
13%	0.69	0.70
14%	0.72	0.75
15%	0.74	0.78
